# The role of the Deki Reader™ in malaria diagnosis, treatment and reporting: findings from an Africare pilot project in Nigeria

**DOI:** 10.1186/s12936-018-2356-8

**Published:** 2018-06-01

**Authors:** Patrick Adah, Omosivie Maduka, Obinna Obasi, Orode Doherty, Susana Oguntoye, Kayla Seadon, Oren Jalon, Nora Zwingerman, Perpetua Uhomoibhi

**Affiliations:** 1 Africare Nigeria, Lagos, Nigeria; 2 Department of Preventive and Social Medicine, College of Health Sciences, University of Port Harcourt, PMB 5323, Port Harcourt, Nigeria; 3 Africare USA, Washington DC, USA; 4 Fio Corporation, Toronto, Canada; 5 National Malaria Elimination Project (NMEP), Federal Ministry of Health, Abuja, Nigeria

## Abstract

**Background:**

The Deki Reader is a diagnostic device used with rapid diagnostic tests (RDTs) and linked to an online database for real-time uploads of patient information and results. This is in contrast to visual interpretation of malaria RDTs recorded on the District Health Information System (DHIS). This paper compares records for use of the Deki Reader with DHIS records of visual interpretation of RDTs.

**Results:**

A total of 4063 patient encounters/tests were recorded on the Deki Reader database between June 1st and December 31st, 2016. These tests were for 2629 persons who presented with fever and had RDT done. In comparison, data from DHIS 2.0 for same period recorded 7201 persons presenting with fever. 2421 out of the 2629 persons (92.1%), received RDT using Deki Reader compared to 6535 out of 7201 persons (90.4%) recorded on DHIS (p = 0.04). From DHIS records, malaria positivity rate was 51.6% (3375 out of 6535 persons) compared to Deki Reader records of 23.6% (572 out of 2421 persons). The difference between these two rates was significant (p < 0.001). The odds ratio (95% CI) for the association between use of Deki Reader and having a positive malaria result was 0.29 (0.26–0.32). DHIS showed that 4008 persons received Artemisinin-based combination therapy (ACT) while 3989 persons tested positive with RDT or microscopy, compared to 691 out of 705 persons (98.0%) using Deki Reader. Finally, Deki Reader identified 618 processing and manufacturers errors with an error rate of 15.3%.

**Conclusion:**

The Deki Reader is likely a useful tool for malaria diagnosis, treatment, and real-time data management. It potentially improves diagnostic quality, reduces wastage in ACT administration and improves data quality.

## Background

Nigeria has a high burden of malaria with all year round transmission and over 97% of its population at risk of infection [1]. The goal of the National Malaria Elimination Programme (NMEP) is “to reduce malaria burden to pre-elimination levels and bring malaria-related mortality to zero” [1]. In line with this goal, three of its seven objectives are to ensure that all persons with suspected malaria who seek care are tested with RDT or microscopy by 2020; all persons with confirmed malaria seen in private or public health facilities, receive prompt treatment with an effective anti-malarial drug by 2020; and, 100% of health facilities report on key malaria indicators routinely by 2020 [1].

The 2010 Nigerian Malaria Indicator Survey (NMIS), put the overall malaria parasite prevalence at 42% detected using microscopy [2]. The 2015 NMIS key indicators showed a reduction in the overall parasite prevalence to 27.4% detected using microscopy and 45.1% detected using the malaria rapid diagnostic test (RDT) [3]. This reduction is likely attributable to an intensification of malaria control and eradication efforts over the period. Underpinning effective implementation of point-of-care (PoC), testing with RDT, is the programmatic paradigm shift from presumptive diagnosis and treatment of all fevers as malaria to a parasite-based diagnosis of malaria with microscopy or RDTs, and treatment with artemisinin-based combination therapy (ACT) in line with the World Health Organization’s 2010 and 2015 guidelines [4, 5].

The role of RDTs in malaria control efforts has been well documented [6–8]. RDTs for malaria began to be available on a large-scale in Nigeria in 2010 [9, 10]. While acceptance has risen in recognition of RDT as a reliable and cost-effective test for parasite-based diagnosis of malaria, there have been challenges. These challenges include concerns about the ability of RDTs to detect low levels of parasitaemia, and the perceived subjectivity involved in the interpretation of the results [6, 8, 11]. These challenges have resulted in instances of rejection of RDT results, and treatment for malaria in the presence of a negative RDT result [1, 9, 10, 12]. In the light of this, innovations which improve the accuracy of RDT based testing and diagnosis will be beneficial to malaria control efforts.

Nigeria’s health system faces significant challenges in data reporting for decision-making. The District Health Information System (DHIS) version 1.4 was rolled out in 2006 with migration to version 2.0 in 2012 [13, 14]. DHIS is used for routine reporting of health information at all levels of healthcare delivery in Nigeria. Malaria programme indicators are integrated into the DHIS platform. Currently, DHIS relies heavily on health workers manually entering data on a daily and monthly basis using the Health Management Information System (HMIS) registers at the facility level. Also, it relies on Local Government Monitoring and Evaluation (M&E) officers’ collating data from facilities and entering it into the electronic DHIS 2.0 platform at the local government level. The concurrent use of manual and electronic reporting increases the likelihood of error. There is also the issue of delays in availability of data for decision-making using the DHIS platform. This is because data from the index month is uploaded at the beginning of the new month at the earliest, and by the middle of the new month at the latest [14–18].

The Deki Reader is an in vitro diagnostic device used with commercially available rapid diagnostic tests and Fionet mobile software (www.fio.com). The Deki Reader provides: step-by-step guidance for performing rapid diagnostic tests, quality checks for rejecting wrongly processed tests, an objective analysis of test results, test-by-test traceability via records uploaded to Fionet, feedback from remote managers using Fionet two-way messaging and configurable workflows for standardizing care delivery and data capture [19].

The Deki Reader has successfully been deployed in Kenya [16], Tanzania [20], and Colombia [21]. In May 2016, Africare, an international NGO deployed 30 Deki Readers and Fionet mobile software in a pilot project to improve testing, diagnosis and reporting of malaria using RDT in Primary Health Care Centres (PHCs) in two states in the Niger Delta region of Nigeria. This paper presents the findings of a comparison between monthly aggregated Fionet records and DHIS records for fever cases, malaria diagnosis, and malaria treatment.

## Methods

This was a retrospective records-based study to compare fever cases, malaria diagnosis and malaria treatment at the study Primary Health Care Facilities (PHCs).

### Study sites and population

Africare piloted the use of the Deki Reader and Fionet technology in two states of the Niger Delta region of Nigeria; Rivers and Akwa-Ibom. These are states where Africare had received funding for project implementation. Thirty Deki Readers were deployed in 30 PHCs across four LGAs: Eket and Ibeno in Akwa Ibom State, and Ogu Bolo and Bonny Island in Rivers State. The 30 PHCs selected where those which had the highest turnover of clients over the preceding 6 months.

The functions of the Deki Reader include:

1. Automated interpretation of RDTs using image analysis software;
2. Digital data capture, using a touch-screen and a simple user interface software;
3. Transmission in real time using local mobile phone network of processed RDT image, diagnostic event data collected, geo-positioning of the device, and date and time stamp to a central database, which is accessible via the internet.

### Description of Deki Reader records

One Deki Reader was deployed to each of the 30 selected PHCs in the two states. Each RDT test done was denoted as a patient encounter. For each patient encounter, patient information, test results and images of each test cassette were recorded in a Deki Reader for real-time upload onto the Fionet database. This database was made available via password access to malaria programme officers, and select Africare staff.

### Description of RDTs used

All pilot facilities use CareStart™ Malaria HRP2 (Pf) brand of RDTs manufactured by AccessBio. These are RDTs that have high specificity and sensitivity for the diagnosis of malaria infection from *Plasmodium falciparum* using whole blood of patients. Results are ready within 20 min.

### Description of comparison data from DHIS

In addition to the use of the Deki Reader to capture patient information and interpret RDT tests, the staff at each pilot facility continued to conduct visual interpretation of RDT results and routinely capture patient information on facility registers and the DHIS platform. From this platform, the relevant data was extracted by Africare programme officers. This was used as comparison data in this study.

### Data collection and analysis

Data was sourced from the DHIS 2.0 and Fionet database and collated in an MS Excel^®^ spreadsheet. WinPepi^®^ statistical software [22] was used to carry out descriptive and inferential statistics to compare data from DHIS and Fionet. Outcome variables for comparison included the proportion of fever cases tested for malaria using RDTs, malaria positivity rates, and proportion of persons with positive RDT treated for malaria with the recommended ACT. The test of significance used was a Chi square to test the homogeneity of the data, with alpha value set at 0.05. Odds ratio was also computed to measure the association between the use of the Deki Reader and malaria positivity rates.

### Ethical considerations

Ethical approval was obtained from the Research Ethics Committee of the University of Port Harcourt. Also, Africare maintains a working agreement with the Rivers and Akwa Ibom State Malaria Elimination Programmes (SMEPs). Data was kept secure, and only authorised personnel within SMEP and Africare were allowed access.

## Results

A total of 4063 patient encounters were recorded on the Deki Reader between the June 1st and December 31st, 2016. Of these, 2629 persons presented with fever. Majority of visits (78.9%) were from patients aged 5 years and above. Almost two-thirds (62.6%) of patient encounters were for women, of which 23.7% were pregnant (Table 1).

**Table 1:** Baseline characteristics of patient encounters^a^ with Deki Reader at pilot PHCs between June and December 2016

Month	Under 5	5 and above	Male	Female	Pregnant	Nonpregnant	Total
June	116	556	261	411	74	337	672
July	70	401	149	322	81	241	471
August	96	563	286	373	81	292	659
September	97	444	178	363	100	263	541
October	163	478	246	395	96	299	641
November	206	444	252	398	82	316	650
December	110	319	147	282	88	194	429
Total	858	3205	1519	2544	602	1942	4063

^a^This refers to entries into the Deki Reader not the number of persons seen in the health facility

### Malaria diagnostic testing

Between June and December 2016, 2629 persons who presented with fever had their details recorded on Deki Readers at the pilot facilities in comparison to 7201 persons with fever whose details were recorded in the facility registers and the DHIS 2.0. This implies that only 36.5% of persons who presented with fever at the health facilities were offered an RDT using Deki Reader assuming DHIS 2.0 data captured all patients.

Among those who presented with fever and were offered an RDT using Deki Reader, a total of 2421 out of 2629 (92.1%) persons, received diagnostic testing for malaria with an RDT. In comparison, 6535 out of 7201 persons (90.4%) were recorded on the DHIS as having received diagnostic testing for malaria using RDT. The overall proportion of cases tested was significantly different between Fionet and DHIS 2.0 data (Chi square = 4.25, *p* value = 0.04). However, analysis of the proportions of individuals tested for each month revealed variation in proportions of persons with fever who received RDT diagnostic testing across months. Data from Fionet had the proportion of cases tested ranging from 88 to 98%. DHIS data had the proportion of cases tested ranging from 84 to 97% (Table 2).

**Table 2:** Comparing Deki Reader and DHIS data for fever cases tested with an RDT in Rivers and Akwa Ibom Pilot facilities

Month	Data from Fionet			Data from DHIS 2.0			Chi square (p-value)
	Number of persons with fever	Fever cases tested with RDT	Proportion of fever cases tested with RDT (%)	Number of persons with fever	Fever cases tested with RDT	Proportion of fever cases tested with RDT (%)	
June	500	466	93.2	1056	904	85.6	8.59 (<.0.001)
July	247	243	98.4	948	825	87.0	26.60 (<.0.001)
August	293	268	91.5	771	727	94.3	2.80 (0.095)
September	368	330	89.7	668	611	91.5	0.92 (0.34)
October	437	423	96.8	1452	1225	84.4	46.63 (<.0.001)
November	452	397	87.8	1245	1195	95.9	37.97 (<.0.001)
December	332	294	88.6	1061	1027	96.8	35.04 (<.0.001)
Total	2629	2421	92.1	7201	6535	90.4	4.25 (0.04)

**Fig. 1:**
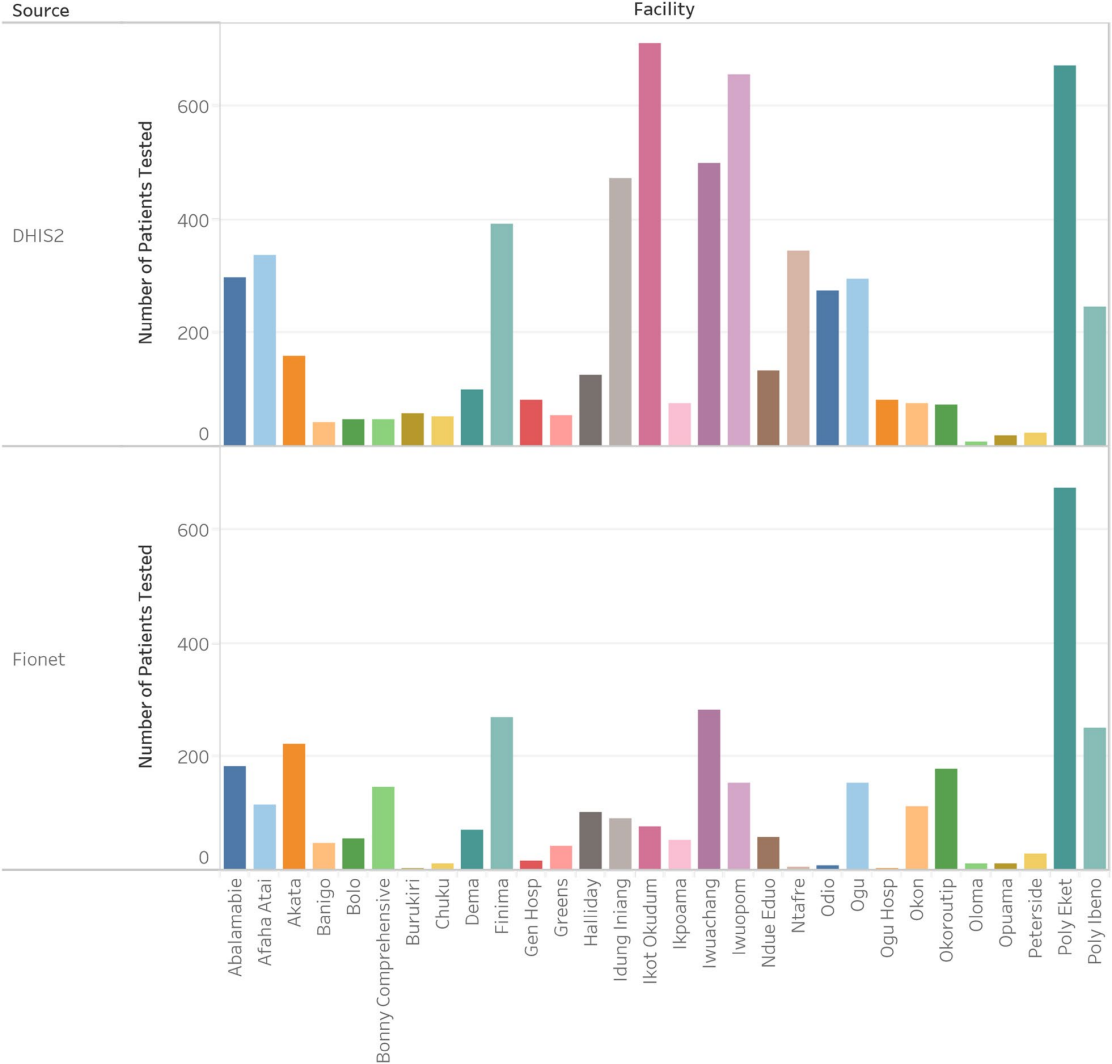
Number of records by facility of the number of patients tested with malaria RDT over the study period

**Fig. 2:**
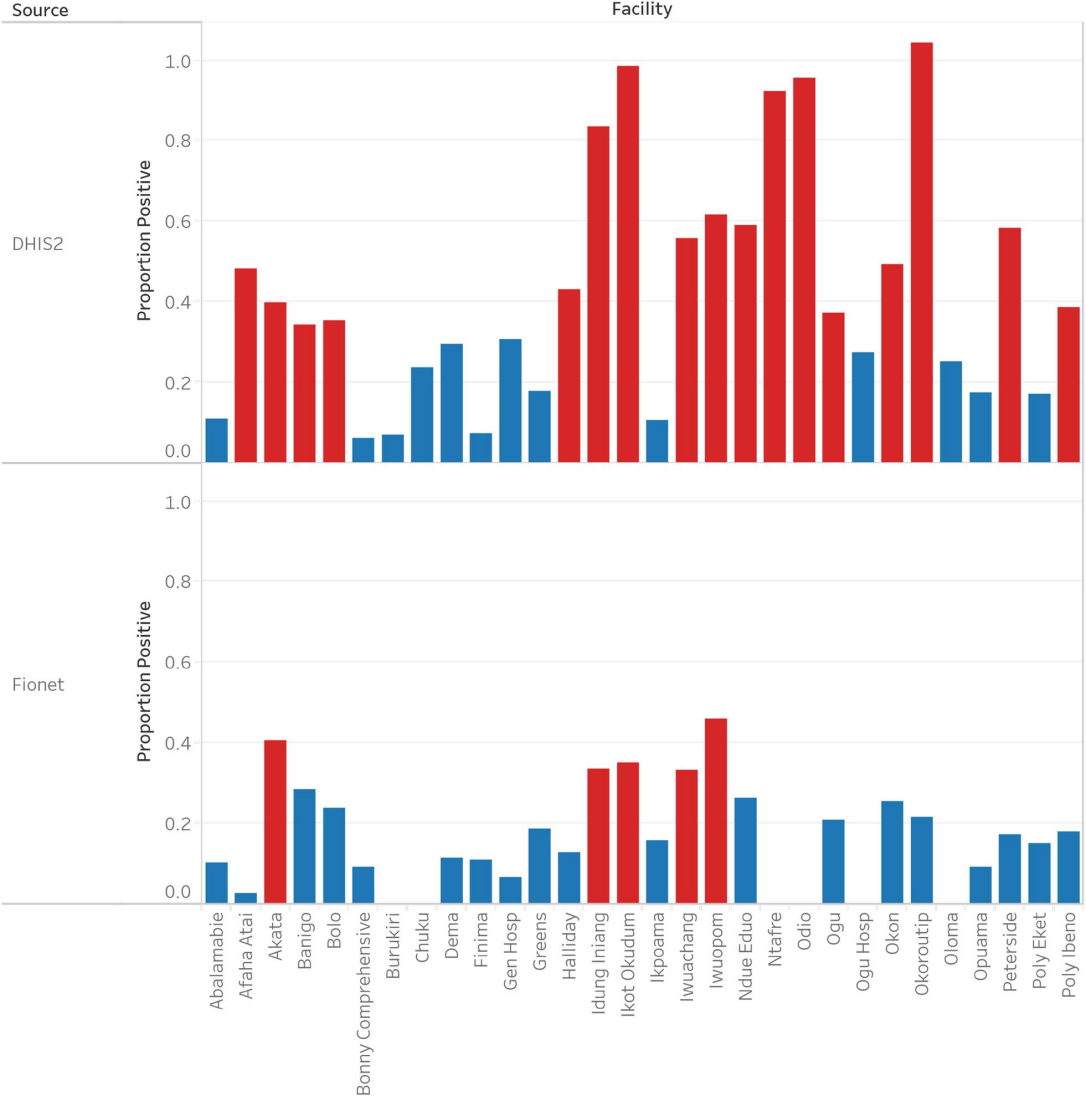
Proportion of patients tested positive by facility. The red bars signify that the positivity rate is above 33%

There was a significant amount of variation in the number of records between the sources, with twenty-five of the thirty facilities having over 20% difference in the number of patients recorded. The number of records by the facility is shown in Fig. 1, while the proportion of patients tested positive by facility is shown in Fig 2.

### Malaria positivity rates

Of the 6535 persons with fever tested for malaria between June and December 2016 recorded on the DHIS, 3375 representing 51.6%, tested positive for malaria. In contrast, Deki Reader records show that 572 persons tested positive out of 2421 with fever, representing a 23.6% malaria positivity rate. The difference between these proportions was significant (Chi square = 562.63, p-value < 0.001). Furthermore, the odds ratio (95% CI) for the association between use of Deki Reader and having a malaria positive result was 0.29 (0.26–0.32). When analysing the data by facility, the DHIS 2 data shows seventeen, out of thirty, facilities had positivity rates over 33% (highlighted in red). On the other hand, Fionet had an overall positivity rate of 23% with only five facilities having positivity rates over 33% (highlighted in red). This indicates that the higher positivity rates observed with DHIS 2.0 was driven by a few facilities reporting high positivity rates (Table 3).

**Table 3:** Comparing the Deki Reader and visual interpretation (from DHIS data) for malaria positivity rates in Rivers and Akwa-Ibom pilot facilities

Month	Data from Deki Reader/Fionet			Data from DHIS 2.0			Chi square (p-value)	Odds ratio (95% CI)
	Fever cases tested with RDT	Positive RDT results	Proportion of positive tests (%)	Fever cases tested with RDT	Positive RDT results	Proportion of positive tests (%)		
June	466	115	24.7	904	474	52.4	96.65 (<.0.001)	0.30 (0.23 to 0.38)
July	243	51	21.0	825	458	55.5	89.71 (<.0.001)	0.21 (0.15 to 0.30)
August	268	83	31.0	727	355	48.8	25.35 (<.0.001)	0.47 (0.34 to 0.64)
September	330	80	24.2	611	249	40.8	25.66 (<.0.001)	0.47 (0.34 to 0.63)
October	423	93	22.0	1225	783	63.9	222.03 (<.0.001)	0.16 (0.12 to 0.21)
November	397	79	19.9	1195	509	42.6	129.37 (<.0.001)	0.22 (0.16 to 0.29)
December	294	71	24.2	1027	547	53.3	77.81 (<.0.001)	0.28 (0.21 to 0.38)
Total	2421	572	23.6	6535	3375	51.6	562.63 (<.0.001)	0.29 (0.26 to 0.32)

### Treatment rates

According to facility-based DHIS records, a total of 4008 persons received ACT between June and December 2016 in the facilities. This translates to 101.0% as proportion of persons testing positive for malaria who received malaria treatment. Furthermore, in June, July, September, and November 2016, more persons received ACT than were tested for malaria using either RDT or microscopy. In contrast, the results from the Deki Reader database show that 690 out of 705 (98.0%) persons who tested positive for malaria using a Deki Reader received ACT (Table 4).

**Table 4:** Comparing Deki Reader and DHIS data for ACT treatment rates in Rivers and Akwa Ibom Pilot Facilities

Month	Data from Fionet			Data from DHIS2.0			
	Positive RDT result	Treated with ACT	Treated with ACT (%)	Positive RDT result	Positive microscopy	Treated with ACT^a^	Treated with ACT (%)
June	135	131	97.4	474	113	613	104.4
July	75	75	100.0	458	128	600	102.4
August	102	98	96.1	355	99	437	96.3
September	105	104	99.0	249	60	336	108.7
October	117	116	99.1	783	82	843	97.5
November	90	89	98.9	509	97	612	101.0
December	81	77	95.1	547	35	567	97.4
Total	705	691	98.0	3375	614	4008	101.0

^a^Describes frequency count of patients who received ACT based on a positive result from RDT or microscopy

### Detecting RDT errors

**Fig. 3:**
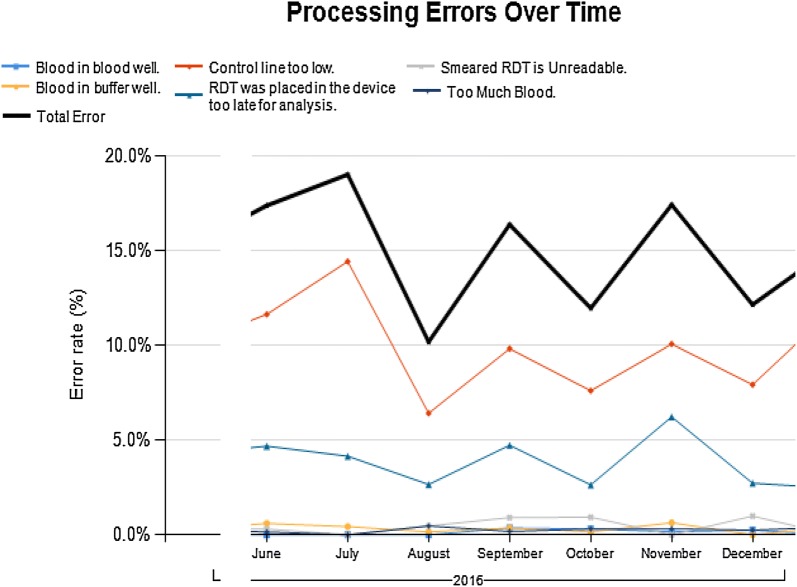
mRDT error rates for June to December 2016 in Akwa Ibom and Rivers States

**Fig. 4:**
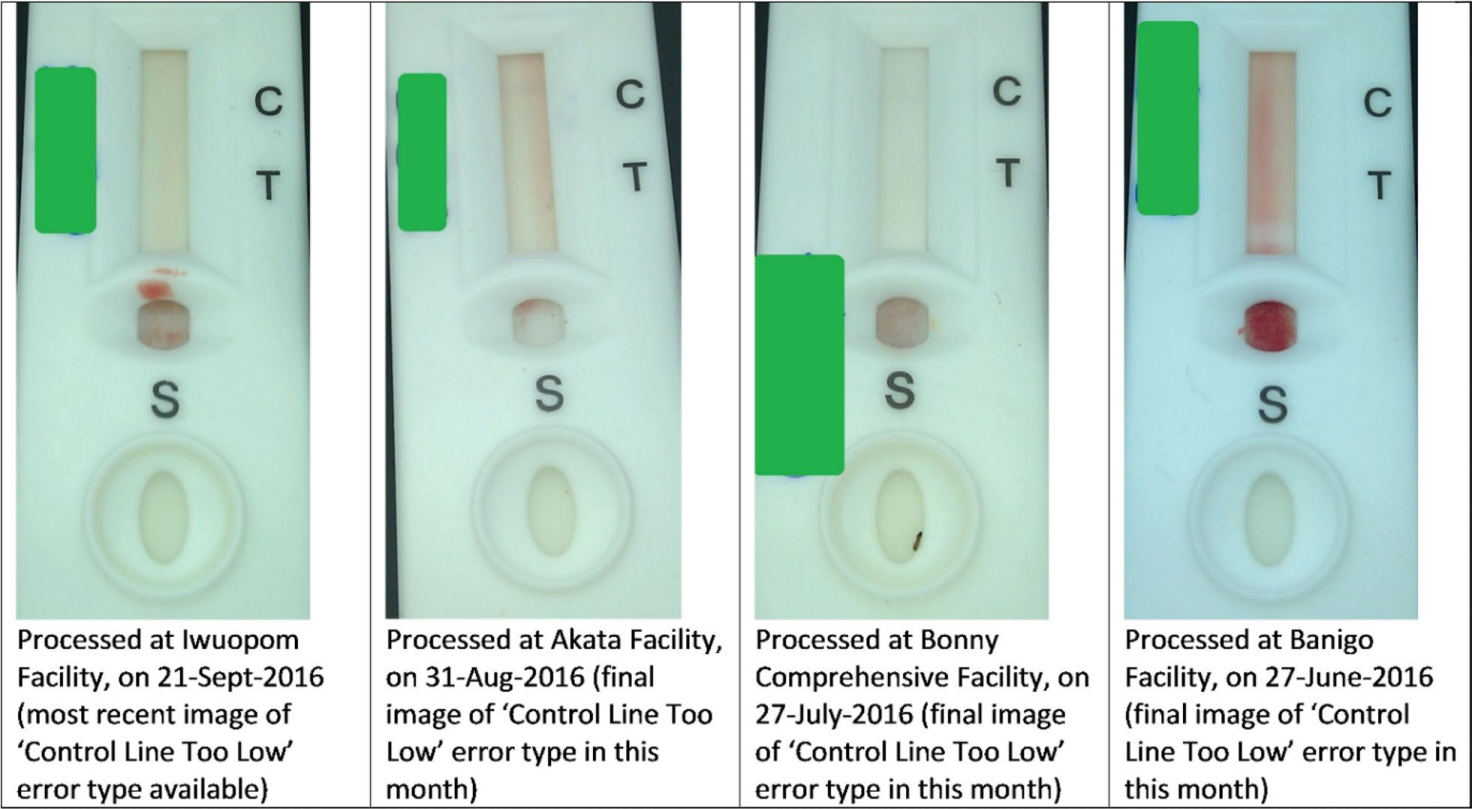
Deki Reader images for the most commonly identified error ‘control line too low’

Between June and December 2016, Deki Reader identified a total of 618 processing and manufacturers’ errors with error rate of 15.3%. The major error identified was “control line too low,” representing 405 errors (10.6%). This error could be due to a manufacturing defect or inadequate amount of buffer being used so that there is not enough buffer to reach the control line. Processing errors were primarily caused by mistakes made by the user of the Deki Reader in processing of RDT such as adding too much blood (1.1%) and not analysing RDTs in the appropriate amount of time (4.5%). Other reasons for these processing errors are described in Fig. 3. Some select Deki Reader images captured from four pilot facilities showing ‘control line too low’ can be seen in Fig. 4.

## Discussion

The results of this study show the potential of the Deki Reader to improve facility-based malaria indicators related to program objectives. Study findings revealed significant disparities between the number of clients recorded via the facility registers for monthly uploading onto the DHIS 2.0 platform and the real-time data of patient encounters generated from entries into the Deki Reader. Only about a third of records entered into the DHIS had RDTs done using Deki Reader. Malaria positivity rates using the Deki Reader was about half of that obtained from the DHIS database with significantly lower odds of having a malaria positive result if the RDT was done with a Deki Reader, compared with RDT results recorded on DHIS 2.0. The positivity rate by facility varied substantially in the DHIS 2.0 data. The number of persons who received ACTs as recorded by DHIS 2.0 was much higher than in the Deki Reader data. There was greater variability in the proportion of persons treated with ACT recorded on DHIS 2.0 data with instances of treatment rates above 100%. Finally, the Deki Reader was useful in identifying various RDT manufacturing and processing errors in over a tenth of RDTs done with the highest error rates coming from no control line on the RDT.

These findings have several implications for facility-based malaria diagnosis, treatment and information management systems. The disparity between the number of consultations and diagnostic tests recorded on Fionet as compared to DHIS may imply that staff are still adjusting to the perceived additional workload of using the Deki Reader or are making selective decisions about for whom to use the Deki Reader. Additionally, each facility had only one Deki Reader with possibly multiple RDT stations. Issues with acceptability of the location of the Deki Reader, unreliable electricity supply, may also be an inhibitor to Deki Reader uptake in these facilities. On the other hand, the observed disparity may be a pointer to issues with DHIS data integrity, especially as DHIS is heavily dependent on retrospective data entry and collation by facility and LGA staff in comparison with the Deki Reader, which uploads records through mobile networks to a web-based portal. This underscores the advantages of automated data generation and upload. Experimental and qualitative studies may be useful in reconciling the observed disparity. Supportive supervision aimed at identifying and resolving bottlenecks to the use of the Deki Reader will also be beneficial.

Study findings showed a disparity between malaria positivity rates from Deki Reader and DHIS data. Furthermore, using the Deki Reader ensured decreased odds of having a positive result for malaria. This points to the Deki Reader device as a potentially useful intervention for improving the accuracy of RDTs. Figures obtained with Deki Reader closely approximate positivity rates obtained using the gold standard of microscopy during the 2015 Malaria Indicator Survey [3]. The ability of the Deki Reader to reduce the likelihood of errors from visual interpretation may be responsible for the lower malaria positivity rates obtained using Deki Reader in comparison to that recorded on DHIS 2.0. This may imply greater effectiveness of diagnosis-based treatment of malaria using Deki Reader.

Study findings relating to the high proportions of persons treated with ACT also provide evidence that staff in the pilot PHCs may be dispensing ACT medicines for malaria treatment to persons who had not been tested for malaria or even those with negative test results. This finding also raises questions relating to the quality of data uploaded to the DHIS. The Deki Reader requires that the post-diagnosis treatment plan be recorded and uploaded in real time. This has the potential to foster the ‘track, test and treat’ policy. Ultimately, this could be a useful tool to reduce ACT wastages and possibly slow the onset of resistance due to irrational use of ACT. The Deki Reader also offers prospects for improving data quality regarding completeness and accuracy. This is because it provides real-time remote supervision through protocol guidance and a two-way messaging system including image capture. In contrast, the current M&E system provides little to no daily oversight for the activities of health workers.

Study findings also reveal the role of Deki Readers in reducing the number of invalid tests arising from manufacturers’ errors or poor technique of health workers. Deki Reader captures and uploads images of each RDT test done. This together with its ability to identify processing errors will aid on-the job-capacity-building activities for health workers. Of note is the particularly high level of manufacturing errors relating to the control line being too low. This points to the need for the programme to review the quality assurance processes for procurement, distribution, and storage of RDTs.

The Deki Reader is a relatively new technology. Only three other studies have been published on the use of the Deki Reader for malaria diagnosis and testing. These studies highlighted several positive effects of using the Deki Reader for malaria diagnosis, treatment, and patient information management including high specificity and sensitivity rates compared to microscopy or PCR [16, 20, 21]. Findings from this research are in agreement with these publications. Research done in 2013 in Tanzania and 2014 in Colombia, showed good performance using the Deki Reader and highlight its “usefulness in the health care sector” [20, 21]. Research done in 2015 in Kenya, found the Deki Reader to be a paperless innovation that brings promise for “improvement in quality control and quality assurance of malaria diagnosis, care and data management” [16].

This study is limited by the retrospective use of aggregated data for comparison. A randomized control trial would have provided stronger evidence. These are areas for future research. More research is also needed to explore health worker perspectives about usefulness, location and challenges with the use of the Deki Reader. This study also did not calculate specificity and sensitivity of the Deki Reader since a recent study had established this [16]. All pilot facilities use CareStart™ brand of RDTs (Fig. 3). As such, study findings relate to Deki Reader interpretation of only these RDT. However other studies in which other brands of RDTs were in use have shown similar findings [16, 20, 21]. Finally, Deki Reader currently creates an entry system that runs parallel to paper-based data collection on HMIS registers for upload to DHIS. There may, therefore, be greater uptake of the Deki Reader when it is the sole system of data capture.

## Conclusion

The Deki Reader is an innovation for evidence-based malaria interventions. This study found significant differences in all malaria program indicators in comparison with DHIS records for visual interpretation of RDTs. Its usefulness applies to malaria diagnosis and treatment as well as real-time management of program-specific data. It may be relevant for improving diagnostic quality, reducing wastage in ACT administration and improving validity of data for decision-making.
